# Occurrence dataset of birds in Sihong Hongze Lake Wetlands National Nature Reserve in China

**DOI:** 10.3897/BDJ.11.e113108

**Published:** 2023-12-01

**Authors:** Huali Hu, Wei Hu, Zheping Xu, Changhu Lu

**Affiliations:** 1 College of Life Science, Nanjing Forestry University, Nanjing, China College of Life Science, Nanjing Forestry University Nanjing China; 2 National Science Library, Chinese Academy of Sciences, Beijing, China National Science Library, Chinese Academy of Sciences Beijing China

**Keywords:** Sihong Hongze Lake, wetland, birds, dataset

## Abstract

**Background:**

Hongze Lake is China’s fourth largest freshwater lake and is also an important habitat for hundreds of thousands of migratory birds on the East Asian-Australian Flyway (EAAF). Sihong Hongze Lake Wetlands National Nature Reserve is located on the northwest of Hongze Lake, Sihong County, Jiangsu Province. The Reserve is a protected large area of natural lake wetlands, marsh wetlands and riverine wetlands and used as a stopover and wintering habitats for migratory birds. Previous studies have conducted bird diversity and temporal-spatial variation in this Reserve, but only for species of Anseriformes. There is still a lack of a comprehensive dataset on the number of bird species and individuals in this Reserve throughout the year. Our study was conducted from July 2020 to June 2021 to observe bird species composition and individual numbers at Sihong Hongze Lake Wetlands National Nature Reserve and provides an occurrence dataset with detailed species and geographic information.

**New information:**

This occurrence dataset is the first public record of birds in Sihong Hongze Lake Wetlands National Nature Reserve for a whole year, which includes the taxonomic information, location information, number, investigation date and endangered level for each species. All data have been published on GBIF.

## Introduction

A lake wetland ecosystem consists of shallow water, marshes, mudflats and sparse grass flats that are natural habitats and feeding grounds for migratory birds and other wetland-dependent wildlife, especially some endangered species (Paszkowski and Tonn 2000, Lyche-Solheim et al. 2013, Wang et al. 2019). The middle and lower reaches of the Yangtze River Basin have the most representative and largest concentration of freshwater lakes in China and provides an important habitat for globally significant numbers of birds (Zhang et al. 2022). However, intensive human activities (agriculture, urbanisation, land reclamation and conversion) and anthropogenic factors (global climatic variation and flooding) have led to the reduction of the lake area and ecological degradation, making the middle and lower reaches of the Yangtze River Basin one of the most endangered areas in China (Cui et al. 2013, Wang X et al. 2020).

Since the 20^th^ century, the Chinese government has taken active measures to protect wetlands. At present, a wetland protection network system has been basically formed with wetland nature reserves as the main body and internationally important wetlands and wetland parks as a combination, which have become an effective measure to protect endangered and rare birds (Zheng et al. 2012). Nature reserves have been established in important lakes, such as Poyang Lake and Dongting Lake and systematic bird research has been carried out in these areas. For example, Debela et al. (2020) have clarified the composition and diversity of the over-wintering aquatic bird community in three nature reserves around Poyang Lake. Yang et al. (2016) have explored the spatiotemporal pattern of bird habitats in Poyang Lake. Zhu et al. (2021) have studied habitat suitability of migratory birds to the water level fluctuations on Dongting Lake.

Hongze Lake is the fourth largest freshwater lake in China and the largest water storage lake along the Eastern Route of the South-North Water Diversion Project (Yang et al. 2021, Zhu et al. 2022). Sihong Hongze Lake Wetlands National Nature Reserve (SHLWNNR) is located at the northwest of Hongze Lake, which is the most completely preserved and representative reserve in China with the breeding of plentiful biological species (Qin et al. 2020). The total area of the Reserve is 49365 hm^2^, the wetland types mainly including lake wetlands, marsh wetlands and river wetlands, accounting for 92.26% of the total area of the Reserve (Ye et al. 2004). In addition, the Reserve is located in a dense intersection area of the East Asian-Australian Flyway (EAAF), which is an important stopover site and overwintering ground for migratory birds and a crucial area for the protection of rare and endangered bird species (Jiang et al. 2017).

Bird studies in SHLWNNR are generally lacking and available reports are not specific and incomplete. For instance, Wang et al. (2014) conducted a comprehensive scientific expedition to the Reserve and recorded 147 bird species, belonging to 15 orders and 47 families. The management office of the Reserve prepared the "2016-2019 Bird Survey Report of Sihong Hongze Lake Wetlands National Nature Reserve", based on the observations and records from 2016 to 2019. From the results of the four-year bird survey, a total of 207 species of birds were recorded, belonging to 14 orders and 53 families. As of July 2021, a total of seven bird observation reports have been collected from the Reserve in the China Bird Report (http://www.birdreport.cn/), belonging to 84 species of birds in 14 orders and 34 families. Wang et al. (2022) discussed the population distribution and interannual variation of Anatidae in the Reserve during the wintering period and showed that there were 24 Anatidae species with the number of recorded species and population increasing year by year. Qin et al. (2020) studied different vegetation planting schemes to promote the protection of typical summer migratory birds (*Chlidoniashybrida* (Pallas, 1811)) and winter migratory birds (*Fulicaatra* Linnaeus, 1758) in the Reserve. However, these studies recorded and published bird data for only some months or for some species and the research of overall bird diversity are from many years ago. Therefore, we conducted a more comprehensive and systematic field survey to publish an occurrence dataset for updating the bird species list and identifying the status of bird resources in the Reserve throughout the year. It also provides basic data for subsequent researchers to conduct bird research and endangered species conservation in the future.

## Sampling methods

### Sampling description

**Figure 1.**
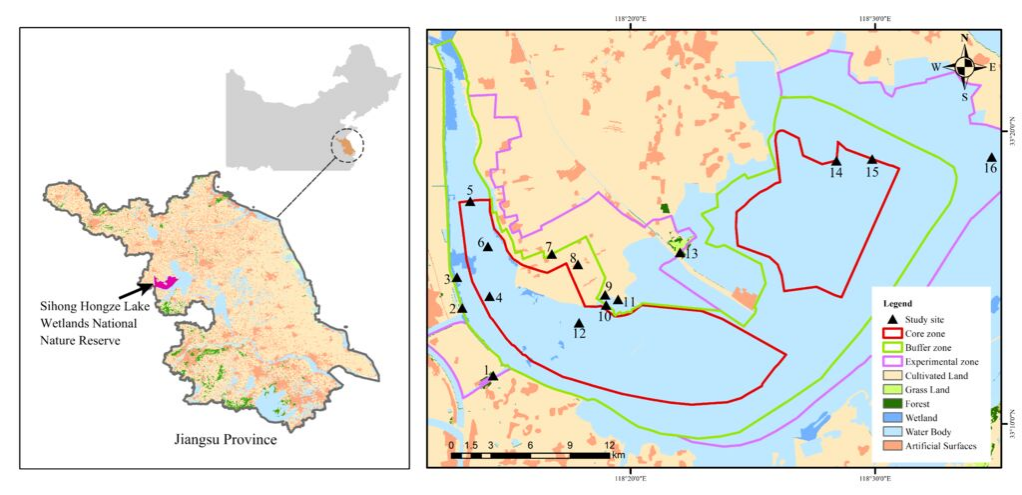
Location of the study sites in Sihong Hongze Lake Wetlands National Nature Reserve.

In this study, the fixed-point observation method was used to record the individual number of bird species in SHLWNNR. A total of 16 study sites were set up, based on habitat characteristics and the accessibility to conduct field surveys in the Reserve and every study site functioned as a fixed point for bird surveys（Fig. 1). The investigation time of the bird survey was generally chosen in the morning or evening in good weather conditions and each survey lasted for 5-7 days. During the bird survey, the investigators used a Nikon 20×60 monocular telescope and 10 × 42 binoculars (Shuntu) to survey each fixed-point and the species and numbers of all birds within the observation range were recorded. The observation range consisted of a circular area with a radius of 1 km and the observation time of each study site was 30-40 minutes. Only the birds flying into the sample area were counted, while the birds flying out of the area were not. The counting method adopted a combination of the accurate counting method and the estimation method, the small number of groups adopting the direct accurate counting method, while the larger number of groups adopted the group statistics method. At the same time, a Canon EOS 70d camera with an EF 100-600 mm f/4.5-5.6L ISII USM lens was used to photograph birds and their habitats in the study sites. The determination of species names and bird classifications was mainly based on *A Checklist on the Classification and Distribution of the Birds of China (Third Edition)* (Zheng 2017). The final dataset was organised according to the Darwin Core format and uploaded to GBIF upon the conclusion of annual surveys（Hu et al. 2023）.

## Geographic coverage

### Description

We downloaded the image of land-cover type from GlobeLand30 (http://www.globallandcover.com/) and drew the investigation scope by using ArcGIS 10.8. A total of 16 study sites were set up, covering all habitat types of the Reserve.

### Coordinates

33.19 N and 33.32 N Latitude; 118.22 E and 118.58 E Longitude.

## Taxonomic coverage

### Description

SHLWNNR is an important habitat for rare and endangered birds and, after a year of bird surveys, a total of 201918 detections for 215 species belonging to 18 orders and 55 families were recorded in this occurrence dataset (Table 1), including 37 species which were listed in the category of national key protected wild animals (National Forestry and Grassland Administration, Ministry of Agriculture and Rural Affairs 2021) and seven species listed in the IUCN Red List as Threatened Species (IUCN 2023). Twenty-five new species were recorded compared to previous records in the Reserve, 11 bird species were not recorded and all of the birds that were not rediscovered were recorded in the last century of history, which may be a function of the distribution of birds in relation to changes in global temperatures. In the category of China’s key protected wildlife; *Aythyabaeri* (Radde, 1863), *Ciconianigra* (Linnaeus, 1758), *Ciconiaboyciana* Swinhoe, 1873, *Plataleaminor* Temminck & Schlegel, 1849 and *Aegypiusmonachus* (Linnaeus, 1766) were ranked as National First-class Protected Wildlife; *Ansercygnoid* (Linnaeus, 1758), *Anseralbifrons* (Scopoli, 1769), *Cygnuscolumbianus* (Ord, 1815), *Aixgalericulata* (Linnaeus, 1758), *Nettapuscoromandelianus* (Gmelin, 1789), *Sibirionettaformosa* (Georgi, 1775), *Mergellusalbellus* (Linnaeus, 1758), *Centropusbengalensis* (Gmelin, 1788), *Hydrophasianuschirurgus* (Scopoli, 1786), *Numeniusarquata* (Linnaeus, 1758), *Platalealeucorodia* Linnaeus, 1758, *Pandionhaliaetus* (Linnaeus, 1758), *Elanuscaeruleus* (Desfontaines, 1789), *Accipiternisus* (Linnaeus, 1758), *Accipitergentilis* (Linnaeus, 1758), *Circusspilonotus* Kaup, 1847, *Circuscyaneus* (Linnaeus, 1766), *Circusmelanoleucos* (Pennant, 1769), *Milvusmigrans* (Boddaert, 1783), *Buteojaponicus* Temminck & Schlegel, 1844, *Otussunia* (Hodgson, 1836), *Glaucidiumcuculoides* (Vigors, 1831), *Athenenoctua* (Scopoli, 1769), *Asioflammeus* (Pontoppidan, 1763), *Falcotinnunculus* Linnaeus, 1758, *Falcoamurensis* Radde, 1863, *Falcoperegrinus* Tunstall, 1771, *Paradoxornisheudei* David, 1872, *Zosteropserythropleurus* Swinhoe, 1863, *Garrulaxcanorus* (Linnaeus, 1758), *Calliopecalliope* (Pallas, 1776) and *Saxicolainsignis* J.E.Gray & G.R.Gray, 1847 were ranked as National Second-class Protected Wildlife. In the IUCN Red List, *Aythyabaeri* (Radde, 1863) was ranked as Critically Endangered (CR); *Ciconiaboyciana* Swinhoe, 1873 and *Plataleaminor* Temminck & Schlegel, 1849 were ranked as Endangered (EN); *Ansercygnoid* (Linnaeus, 1758), *Aythyaferina* (Linnaeus, 1758), *Saxicolainsignis* J.E.Gray & G.R.Gray, 1847 and *Emberizarustica* Pallas, 1776 were ranked as Vulnerable (VU).

## Temporal coverage

**Data range:** 2020-7-20 – 2021-6-24.

### Notes

The specific dates of this period were: 2020-07-20~2020-07-24； 2020-08-21~2020-08-25； 2020-09-20~2020-09-25； 2020-10-21~2020-10-25; 2020-11-17~2020-11-23； 2020-12-19~2020-12-24； 2021-01-22~2021-01-27； 2021-02-22~2021-02-28； 2021-03-19~2021-03-25； 2021-04-21~2021-04-26； 2021-05-20~2021-05-25； 2021-06-19~2021-06-24.

## Usage licence

### Usage licence

Creative Commons Public Domain Waiver (CC-Zero)

### IP rights notes

Creative Commons Attribution Non Commercial (CC-BY-NC) 4.0 License

## Data resources

### Data package title

Occurrence dataset of birds in the Sihong Hongze Lake Wetland National Nature Reserve, Jiangsu, China

### Resource link

http://www.gbif.org/dataset/c5ccfa7e-eda3-47e3-9652-ab1104576125

### Alternative identifiers

https://doi.org/10.15468/p2h2f9; http://www.gbifchina.org.cn/resource?r=sihong_hongze_lake_bird

### Number of data sets

1

### Data set 1.

#### Data set name

Occurrence dataset of birds in the Sihong Hongze Lake Wetland National Nature Reserve, Jiangsu, China

#### Data format

Darwin Core Archive format

#### Download URL

http://www.gbifchina.org.cn/resource?r=sihong_hongze_lake_bird&v=1.2

#### Description

Our occurrence data contains 34 column labels. All occurrence records are georeferenced.

**Data set 1.** 

Column label	Column description
eventID (Event Core, Occurrence Extension)	An identifier for the set of information associated with an Event (something that occurs at a place and time). May be a global unique identifier or an identifier specific to the dataset.
parentEventID (Event Core)	An identifier for the broader Event that groups this and potentially other Events.
eventDate (Event Core)	The date when the event was recorded.
samplingProtocol (Event Core)	The names of, references to, or descriptions of the methods or protocols used during an Event.
samplingEffort (Event Core)	The amount of effort expended during an Event.
sampleSizeValue (Event Core)	A numeric value for a measurement of the size (time duration, length, area or volume) of a sample in a sampling event.
sampleSizeUnit (Event Core)	The unit of measurement of the size (time duration, length, area or volume) of a sample in a sampling event.
decimalLongitude (Event Core)	The geographic longitude of the geographic centre of a Location.
decimalLatitude (Event Core)	The geographic latitude of the geographic centre of a Location.
geodeticDatum (Event Core)	The ellipsoid, geodetic datum or spatial reference system (SRS), upon which the geographic coordinates given in decimalLatitude and decimalLongitude are based.
countryCode (Event Core)	The standard code for the country in which the Location occurs.
country (Event Core)	The name of the country in which the Location occurs.
stateProvince (Event Core)	The name of the next smaller administrative region than country (state, province, canton, department, region etc.) in which the Location occurs.
county (Event Core)	The full, unabbreviated name of the next smaller administrative region than stateProvince (county, shire, department etc.) in which the Location occurs.
locality (Event Core)	The specific description of the place.
coordinateUncertaintyInMetres (Event Core)	The horizontal distance (in metres) from the given decimalLatitude and decimalLongitude describing the smallest circle containing the whole of the Location. Leave the value empty if the uncertainty is unknown, cannot be estimated or is not applicable (because there are no coordinates). Zero is not a valid value for this term.
occurrenceID (Occurrence Extension)	An identifier for the bird occurrence.
basisOfRecord (Occurrence Extension)	The specific nature of the data record.
recordedBy (Occurrence Extension)	A list (concatenated and separated) of names of people, groups or organisations responsible for recording the original Occurrence. The primary collector or observer, especially one who applies a personal identifier (recordNumber), should be listed first.
individualCount (Occurrence Extension)	The number of individuals present at the time of the Occurrence.
organismQuantity (Occurrence Extension)	A number or enumeration value for the quantity of organisms.
organismQuantityType (Occurrence Extension)	The type of quantification system used for the quantity of organisms.
occurrenceStatus (Occurrence Extension)	A statement about the presence or absence of a Taxon at a Location.
scientificName (Occurrence Extension)	The full scientific name.
scientificNameAuthorship (Occurrence Extension)	The authorship information for the scientificName formatted according to the conventions of the applicable nomenclaturalCode.
kingdom (Occurrence Extension)	The full scientific name of the kingdom in which the taxon is classified.
phylum (Occurrence Extension)	The full scientific name of the phylum in which the taxon is classified.
class (Occurrence Extension)	The full scientific name of the class in which the taxon is classified.
order (Occurrence Extension)	The full scientific name of the order in which the taxon is classified.
family (Occurrence Extension)	The full scientific name of the family in which the taxon is classified.
genus (Occurrence Extension)	The full scientific name of the genus in which the taxon is classified.
taxonRank (Occurrence Extension)	The taxonomic rank of the most specific name in the scientificName as it appears in the original record.
ownerInstitutionCode (Occurrence Extension)	The name (or acronym) in use by the institution having ownership of the object(s) or information referred to in the record.
dynamicProperties (Occurrence Extension)	A list of threatened level about the record according to the IUCN Red List of Threatened Species (Version 2022-2). Meant to provide a mechanism for structured content.

## Figures and Tables

**Table 1.** Bird list in Sihong Hongze Lake Wetlands National Nature Reserve.

Rank	Order	Family	Scientific Name	The number of individual birds observed
1	Galliformes	Phasianidae	*Coturnixjaponica* Temminck & Schlegel, 1849	3
2	Galliformes	Phasianidae	*Phasianuscolchicus* Linnaeus, 1758	163
3	Anseriformes	Anatidae	*Ansercygnoid* (Linnaeus, 1758)	42
4	Anseriformes	Anatidae	*Anserfabalis* (Latham, 1787)	208
5	Anseriformes	Anatidae	*Anseranser* (Linnaeus, 1758)	38
6	Anseriformes	Anatidae	*Anseralbifrons* (Scopoli, 1769)	4
7	Anseriformes	Anatidae	*Cygnuscolumbianus* (Ord, 1815)	353
8	Anseriformes	Anatidae	*Tadornatadorna* (Linnaeus, 1758)	22
9	Anseriformes	Anatidae	*Tadornaferruginea* (Pallas, 1764)	4
10	Anseriformes	Anatidae	*Aixgalericulata* (Linnaeus, 1758)	8
11	Anseriformes	Anatidae	*Nettapuscoromandelianus* (Gmelin, 1789)	1
12	Anseriformes	Anatidae	*Marecastrepera* (Linnaeus, 1758)	4856
13	Anseriformes	Anatidae	*Marecafalcata* (Georgi, 1775)	6061
14	Anseriformes	Anatidae	*Marecapenelope* (Linnaeus, 1758)	252
15	Anseriformes	Anatidae	*Anasplatyrhynchos* Linnaeus, 1758	7358
16	Anseriformes	Anatidae	*Anaszonorhyncha* Swinhoe, 1866	11281
17	Anseriformes	Anatidae	*Anasacuta* Linnaeus, 1758	899
18	Anseriformes	Anatidae	*Anascrecca* Linnaeus, 1758	7164
19	Anseriformes	Anatidae	*Spatulaclypeata* (Linnaeus, 1758)	1106
20	Anseriformes	Anatidae	*Spatulaquerquedula* (Linnaeus, 1758)	1134
21	Anseriformes	Anatidae	*Sibirionettaformosa* (Georgi, 1775)	7813
22	Anseriformes	Anatidae	*Aythyaferina* (Linnaeus, 1758)	933
23	Anseriformes	Anatidae	*Aythyabaeri* (Radde, 1863)	17
24	Anseriformes	Anatidae	*Aythyanyroca* (Guldenstadt, 1770)	714
25	Anseriformes	Anatidae	*Aythyafuligula* (Linnaeus, 1758)	76
26	Anseriformes	Anatidae	*Mergellusalbellus* (Linnaeus, 1758)	9
27	Anseriformes	Anatidae	*Mergusmerganser* Linnaeus, 1758	2
28	Podicipediformes	Podicipedidae	*Tachybaptusruficollis* (Pallas, 1764)	1246
29	Podicipediformes	Podicipedidae	*Podicepscristatus* (Linnaeus, 1758)	169
30	Columbiformes	Columbidae	*Streptopeliaorientalis* (Latham, 1790)	1888
31	Columbiformes	Columbidae	*Streptopeliadecaocto* (Frivaldszky, 1838)	9
32	Columbiformes	Columbidae	*Streptopeliatranquebarica* (Hermann, 1804)	93
33	Columbiformes	Columbidae	*Streptopeliachinensis* (Scopoli, 1786)	300
34	Caprimulgiformes	Caprimulgidae	*Caprimulgusindicus* Latham, 1790	3
35	Caprimulgiformes	Apodidae	*Apuspacificus* (Latham, 1801)	23
36	Cuculiformes	Cuculidae	*Centropusbengalensis* (Gmelin, 1788)	3
37	Cuculiformes	Cuculidae	*Eudynamysscolopaceus* (Linnaeus, 1758)	20
38	Cuculiformes	Cuculidae	*Hierococcyxsparverioides* (Vigors, 1832)	10
39	Cuculiformes	Cuculidae	*Cuculusmicropterus* Gould, 1838	14
40	Cuculiformes	Cuculidae	*Cuculuscanorus* Linnaeus, 1758	90
41	Gruiformes	Rallidae	*Rallusindicus* Blyth, 1849	2
42	Gruiformes	Rallidae	*Amaurornisphoenicurus* (Pennant, 1769)	15
43	Gruiformes	Rallidae	*Gallinulachloropus* (Linnaeus, 1758)	1185
44	Gruiformes	Rallidae	*Fulicaatra* Linnaeus, 1758	99216
45	Charadriiformes	Recurvirostridae	*Himantopushimantopus* (Linnaeus, 1758)	50
46	Charadriiformes	Recurvirostridae	*Recurvirostraavosetta* Linnaeus, 1758	8
47	Charadriiformes	Charadriidae	*Vanellusvanellus* (Linnaeus, 1758)	70
48	Charadriiformes	Charadriidae	*Vanelluscinereus* (Blyth, 1842)	55
49	Charadriiformes	Charadriidae	*Pluvialisfulva* (Gmelin, 1789)	2
50	Charadriiformes	Charadriidae	*Charadriusdubius* Scopoli, 1786	20
51	Charadriiformes	Charadriidae	*Charadriusalexandrinus* Linnaeus, 1758	4
52	Charadriiformes	Charadriidae	*Charadriusleschenaultii* Lesson, 1826	1
53	Charadriiformes	Rostratulidae	*Rostratulabenghalensis* (Linnaeus, 1758)	5
54	Charadriiformes	Jacanidae	*Hydrophasianuschirurgus* (Scopoli, 1786)	79
55	Charadriiformes	Scolopacidae	*Gallinagostenura* (Bonaparte, 1831)	2
56	Charadriiformes	Scolopacidae	*Gallinagogallinago* (Linnaeus, 1758)	30
57	Charadriiformes	Scolopacidae	*Limosalimosa* (Linnaeus, 1758)	2
58	Charadriiformes	Scolopacidae	*Numeniusarquata* (Linnaeus, 1758)	10
59	Charadriiformes	Scolopacidae	*Tringaerythropus* (Pallas, 1764)	46
60	Charadriiformes	Scolopacidae	*Tringatotanus* (Linnaeus, 1758)	91
61	Charadriiformes	Scolopacidae	*Tringastagnatilis* (Bechstein, 1803)	1
62	Charadriiformes	Scolopacidae	*Tringanebularia* (Gunnerus, 1767)	22
63	Charadriiformes	Scolopacidae	*Tringaochropus* Linnaeus, 1758	43
64	Charadriiformes	Scolopacidae	*Tringaglareola* Linnaeus, 1758	4
65	Charadriiformes	Scolopacidae	*Actitishypoleucos* (Linnaeus, 1758)	17
66	Charadriiformes	Scolopacidae	*Calidrisalba* (Pallas, 1764)	6
67	Charadriiformes	Glareolidae	*Glareolamaldivarum* J.R.Forster, 1795	2
68	Charadriiformes	Laridae	*Chroicocephalusridibundus* (Linnaeus, 1766)	225
69	Charadriiformes	Laridae	*Larussmithsonianus* Coues, 1862	1
70	Charadriiformes	Laridae	*Sternulaalbifrons* (Pallas, 1764)	2
71	Charadriiformes	Laridae	*Sternahirundo* Linnaeus, 1758	36
72	Charadriiformes	Laridae	*Chlidoniashybrida* (Pallas, 1811)	5499
73	Charadriiformes	Laridae	*Chlidoniasleucopterus* (Temminck, 1815)	17
74	Ciconiiformes	Ciconiidae	*Ciconianigra* (Linnaeus, 1758)	1
75	Ciconiiformes	Ciconiidae	*Ciconiaboyciana* Swinhoe, 1873	37
76	Suliformes	Phalacrocoracidae	*Phalacrocoraxcarbo* (Linnaeus, 1758)	158
77	Pelecaniformes	Threskiornithidae	*Platalealeucorodia* Linnaeus, 1758	115
78	Pelecaniformes	Threskiornithidae	*Plataleaminor* Temminck & Schlegel, 1849	1
79	Pelecaniformes	Ardeidae	*Botaurusstellaris* (Linnaeus, 1758)	14
80	Pelecaniformes	*Ardeidae*	*Ixobrychussinensis (Gmelin, 1789)*	160
81	Pelecaniformes	Ardeidae	*Ixobrychuscinnamomeus* (Gmelin, 1789)	1
82	Pelecaniformes	Ardeidae	*Ixobrychusflavicollis* (Latham, 1790)	2
83	Pelecaniformes	Ardeidae	*Nycticoraxnycticorax* (Linnaeus, 1758)	2503
84	Pelecaniformes	Ardeidae	*Ardeolabacchus* (Bonaparte, 1855)	2010
85	Pelecaniformes	Ardeidae	*Bubulcusibis* (Linnaeus, 1758)	2443
86	Pelecaniformes	Ardeidae	*Ardeacinerea* Linnaeus, 1758	764
87	Pelecaniformes	Ardeidae	*Ardeapurpurea* Linnaeus, 1766	117
88	Pelecaniformes	Ardeidae	*Ardeaalba* Linnaeus, 1758	1044
89	Pelecaniformes	Ardeidae	*Ardeaintermedia* Wagler, 1829	3128
90	Pelecaniformes	Ardeidae	*Egrettagarzetta* (Linnaeus, 1766)	5064
91	Accipitriformes	Pandionidae	*Pandionhaliaetus* (Linnaeus, 1758)	5
92	Accipitriformes	Accipitridae	*Elanuscaeruleus* (Desfontaines, 1789)	17
93	Accipitriformes	Accipitridae	*Aegypiusmonachus* (Linnaeus, 1766)	1
94	Accipitriformes	Accipitridae	*Accipiternisus* (Linnaeus, 1758)	2
95	Accipitriformes	Accipitridae	*Accipitergentilis* (Linnaeus, 1758)	1
96	Accipitriformes	Accipitridae	*Circusspilonotus* Kaup, 1847	20
97	Accipitriformes	Accipitridae	*Circuscyaneus* (Linnaeus, 1766)	16
98	Accipitriformes	Accipitridae	*Circusmelanoleucos* (Pennant, 1769)	2
99	Accipitriformes	Accipitridae	*Milvusmigrans* (Boddaert, 1783)	2
100	Accipitriformes	Accipitridae	*Buteojaponicus* Temminck & Schlegel, 1844	8
101	Strigiformes	Strigidae	*Otussunia* (Hodgson, 1836)	1
102	Strigiformes	Strigidae	*Glaucidiumcuculoides* (Vigors, 1831)	3
103	Strigiformes	Strigidae	*Athenenoctua* (Scopoli, 1769)	1
104	Strigiformes	Strigidae	*Asioflammeus* (Pontoppidan, 1763)	1
105	Bucerotiformes	Upupidae	*Upupaepops* Linnaeus, 1758	42
106	Coraciiformes	Coraciidae	*Eurystomusorientalis* (Linnaeus, 1766)	1
107	Coraciiformes	Alcedinidae	*Halcyonpileata* (Boddaert, 1783)	1
108	Coraciiformes	Alcedinidae	*Alcedoatthis* (Linnaeus, 1758)	111
109	Coraciiformes	Alcedinidae	*Cerylerudis* (Linnaeus, 1758)	38
110	Piciformes	Picidae	*Jynxtorquilla* Linnaeus, 1758	1
111	Piciformes	Picidae	*Dendrocopos* canicapillus (Blyth, 1845)	45
112	Piciformes	Picidae	*Dendrocoposmajor* (Linnaeus, 1758)	84
113	Piciformes	Picidae	*Picuscanus* J.F.Gmelin, 1788	77
114	Falconiformes	Falconidae	*Falcotinnunculus* Linnaeus, 1758	8
115	Falconiformes	Falconidae	*Falcoamurensis* Radde, 1863	9
116	Falconiformes	Falconidae	*Falcoperegrinus* Tunstall, 1771	7
117	Passeriformes	Oriolidae	*Orioluschinensis* Linnaeus, 1766	91
118	Passeriformes	Campephagidae	*Lalagemelaschistos* (Hodgson, 1836)	12
119	Passeriformes	Dicruridae	*Dicrurusmacrocercus* Vieillot, 1817	525
120	Passeriformes	Dicruridae	*Dicrurusleucophaeus* Vieillot, 1817	5
121	Passeriformes	Monarchidae	*Terpsiphoneincei* (Gould, 1852)	6
122	Passeriformes	Laniidae	*Laniustigrinus* Drapiez, 1828	12
123	Passeriformes	Laniidae	*Laniusbucephalus* Temminck & Schlegel, 1845	8
124	Passeriformes	Laniidae	*Laniuscristatus* Linnaeus, 1758	314
125	Passeriformes	Laniidae	*Laniusschach* Linnaeus, 1758	10
126	Passeriformes	Laniidae	*Laniussphenocercus* Cabanis, 1873	306
127	Passeriformes	Corvidae	*Cyanopicacyanus* (Pallas, 1776)	244
128	Passeriformes	Corvidae	*Dendrocittaformosae* Swinhoe, 1863	3
129	Passeriformes	Corvidae	*Picapica* (Linnaeus, 1758)	797
130	Passeriformes	Corvidae	*Corvuscorone* Linnaeus, 1758	2
131	Passeriformes	Paridae	*Pardaliparusvenustulus* (Swinhoe, 1870)	11
132	Passeriformes	Paridae	*Paruscinereus* Vieillot, 1818	143
133	Passeriformes	Remizidae	*Remizconsobrinus* (Swinhoe, 1870)	50
134	Passeriformes	Alaudidae	*Alaudagulgula* Franklin, 1831	151
135	Passeriformes	Cisticolidae	*Cisticolajuncidis* (Rafinesque, 1810)	21
136	Passeriformes	Cisticolidae	*Priniainornata* Sykes, 1832	26
137	Passeriformes	Acrocephalidae	*Acrocephalusorientalis* (Temminck & Schlegel, 1847)	131
138	Passeriformes	Acrocephalidae	*Acrocephalusbistrigiceps* Swinhoe, 1860	12
139	Passeriformes	Hirundinidae	*Ripariariparia* (Linnaeus, 1758)	463
140	Passeriformes	Hirundinidae	*Hirundorustica* Linnaeus, 1758	2009
141	Passeriformes	Hirundinidae	*Cecropisdaurica* (Laxmann, 1769)	1019
142	Passeriformes	Pycnonotidae	*Spizixossemitorques* Swinhoe, 1861	17
143	Passeriformes	Pycnonotidae	*Pycnonotusxanthorrhous* Anderson, 1869	1
144	Passeriformes	Pycnonotidae	*Pycnonotussinensis* (Gmelin, 1789)	3421
145	Passeriformes	Phylloscopidae	*Phylloscopusfuscatus* (Blyth, 1842)	67
146	Passeriformes	Phylloscopidae	*Phylloscopusproregulus* (Pallas, 1811)	156
147	Passeriformes	Phylloscopidae	*Phylloscopusinornatus* (Blyth, 1842)	327
148	Passeriformes	Phylloscopidae	*Phylloscopusborealis* (J.H.Blasius, 1858)	12
149	Passeriformes	Phylloscopidae	*Phylloscopustenellipes* Swinhoe, 1860	4
150	Passeriformes	Phylloscopidae	*Phylloscopuscoronatus* (Temminck & Schlegel, 1847)	2
151	Passeriformes	Cettiidae	*Hororniscanturians* (Swinhoe, 1860)	42
152	Passeriformes	Cettiidae	*Horornisfortipes* Hodgson, 1845	6
153	Passeriformes	Cettiidae	*Urosphenasquameiceps* (Swinhoe, 1863)	1
154	Passeriformes	Aegithalidae	*Aegithalosglaucogularis* (Moore, 1855)	358
155	Passeriformes	Aegithalidae	*Aegithalosconcinnus* (Gould, 1855)	4
156	Passeriformes	Sylviidae	*Sinosuthorawebbiana* (Gould, 1852)	1918
157	Passeriformes	Sylviidae	*Paradoxornisheudei* David, 1872	131
158	Passeriformes	Zosteropidae	*Zosteropserythropleurus* Swinhoe, 1863	14
159	Passeriformes	Zosteropidae	*Zosteropsjaponicus* Temminck & Schlegel, 1845	127
160	Passeriformes	Leiothrichidae	*Garrulaxcanorus* (Linnaeus, 1758)	2
161	Passeriformes	Leiothrichidae	*Garrulaxperspicillatus* (Gmelin, 1789)	14
162	Passeriformes	Sturnidae	*Acridotherescristatellus* (Linnaeus, 1758)	47
163	Passeriformes	Sturnidae	*Spodiopsarsericeus* (Gmelin, 1789)	789
164	Passeriformes	Sturnidae	*Spodiopsarcineraceus* (Temminck, 1835)	2756
165	Passeriformes	Sturnidae	*Gracupicanigricollis* (Paykull, 1807)	4
166	Passeriformes	Turdidae	*Geokichlasibirica* (Pallas, 1776)	1
167	Passeriformes	Turdidae	*Zootheraaurea* (Holandre, 1825)	2
168	Passeriformes	Turdidae	*Turdushortulorum* P.L.Sclater, 1863	24
169	Passeriformes	Turdidae	*Turduscardis* Temminck, 1831	8
170	Passeriformes	Turdidae	*Turdusmandarinus* Bonaparte, 1850	648
171	Passeriformes	Turdidae	*Turduspallidus* Gmelin, 1789	2
172	Passeriformes	Turdidae	*Turdusnaumanni* Temminck, 1820	19
173	Passeriformes	Turdidae	*Turduseunomus* Temminck, 1831	50
174	Passeriformes	Muscicapidae	*Larvivoracyane* (Pallas, 1776)	1
175	Passeriformes	Muscicapidae	*Calliopecalliope* (Pallas, 1776)	1
176	Passeriformes	Muscicapidae	*Tarsigercyanurus* (Pallas, 1773)	37
177	Passeriformes	Muscicapidae	*Copsychussaularis* (Linnaeus, 1758)	2
178	Passeriformes	Muscicapidae	*Phoenicurusauroreus* (Pallas, 1776)	159
179	Passeriformes	Muscicapidae	*Rhyacornisfuliginosa* (Vigors, 1831)	1
180	Passeriformes	Muscicapidae	*Myophonuscaeruleus* (Scopoli, 1786)	2
181	Passeriformes	Muscicapidae	*Saxicolainsignis* J.E.Gray & G.R.Gray, 1847	1
182	Passeriformes	Muscicapidae	*Saxicolamaurus* (Pallas, 1773)	2
183	Passeriformes	Muscicapidae	*Muscicapasibirica* Gmelin, 1789	5
184	Passeriformes	Muscicapidae	*Muscicapadauurica* Pallas, 1811	24
185	Passeriformes	Muscicapidae	*Ficedulazanthopygia* (Hay, 1845)	3
186	Passeriformes	Muscicapidae	*Ficedulanarcissina* (Temminck, 1836)	2
187	Passeriformes	Muscicapidae	*Ficedulamugimaki* (Temminck, 1836)	3
188	Passeriformes	Muscicapidae	*Ficedulaalbicilla* (Pallas, 1811)	1
189	Passeriformes	Muscicapidae	*Cyanoptilacyanomelana* (Temminck, 1829)	1
190	Passeriformes	Bombycillidae	*Bombycillajaponica* (Siebold, 1824)	25
191	Passeriformes	Estrildidae	*Lonchurastriata* (Linnaeus, 1766)	37
192	Passeriformes	Passeridae	*Passercinnamomeus* (Temminck, 1836)	1
193	Passeriformes	Passeridae	*Passermontanus* (Linnaeus, 1758)	869
194	Passeriformes	Motacillidae	*Dendronanthusindicus* (Gmelin, 1789)	1
195	Passeriformes	Motacillidae	*Motacillatschutschensis* Gmelin, 1789	8
196	Passeriformes	Motacillidae	*Motacillacinerea* Tunstall, 1771	200
197	Passeriformes	Motacillidae	*Motacillaalba* Linnaeus, 1758	190
198	Passeriformes	Motacillidae	*Anthusrichardi* Vieillot, 1818	1
199	Passeriformes	Motacillidae	*Anthushodgsoni* Richmond, 1907	215
200	Passeriformes	Motacillidae	*Anthuscervinus* (Pallas, 1811)	1
201	Passeriformes	Motacillidae	*Anthusspinoletta* (Linnaeus, 1758)	8
202	Passeriformes	Fringillidae	*Fringillamontifringilla* Linnaeus, 1758	257
203	Passeriformes	Fringillidae	*Eophonamigratoria* Hartert, 1903	1779
204	Passeriformes	Fringillidae	*Eophonapersonata* (Temminck & Schlegel, 1848)	44
205	Passeriformes	Fringillidae	*Chlorissinica* (Linnaeus, 1766)	72
206	Passeriformes	Fringillidae	*Spinusspinus* (Linnaeus, 1758)	213
207	Passeriformes	Emberizidae	*Emberizacioides* vonJ.F.Brandt, 1843	1
208	Passeriformes	Emberizidae	*Emberizatristrami* Swinhoe, 1870	55
209	Passeriformes	Emberizidae	*Emberizapusilla* Pallas, 1776	204
210	Passeriformes	Emberizidae	*Emberizachrysophrys* Pallas, 1776	40
211	Passeriformes	Emberizidae	*Emberizarustica* Pallas, 1776	132
212	Passeriformes	Emberizidae	*Emberizaelegans* Temminck, 1836	221
213	Passeriformes	Emberizidae	*Emberizaspodocephala* Pallas, 1776	499
214	Passeriformes	Emberizidae	*Emberizapallasi* (Cabanis, 1851)	25
215	Passeriformes	Emberizidae	*Emberizaschoeniclus* (Linnaeus, 1758)	7
